# Educational Interventions to Reduce Prescription and Dispensing of Antibiotics in Primary Care: A Systematic Review of Economic Impact

**DOI:** 10.3390/antibiotics11091186

**Published:** 2022-09-02

**Authors:** Vânia Rocha, Marta Estrela, Vanessa Neto, Fátima Roque, Adolfo Figueiras, Maria Teresa Herdeiro

**Affiliations:** 1 Institute of Biomedicine (iBiMED), Department of Medical Sciences, University of Aveiro, 3810-193 Aveiro, Portugal; 2 Research Unit for Inland Development, Polytechnic of Guarda (UDI-IPG), 6300-559 Guarda, Portugal; 3 Health Sciences Research Centre, University of Beira Interior (CICS-UBI), 6201-001 Covilhã, Portugal; 4 Department of Preventive Medicine and Public Health, University of Santiago de Compostela, 15702 Santiago de Compostela, Spain; 5 Health Research Institute of Santiago de Compostela (IDIS), 15706 Santiago de Compostela, Spain; 6 Consortium for Biomedical Research in Epidemiology and Public Health (CIBER Epidemiology and Public Health—CIBERESP), 28001 Madrid, Spain

**Keywords:** review, microbial drug-resistance, antibiotic prescription, primary healthcare, educational interventions, economic impact

## Abstract

Antibiotic resistance remains a crucial global public health problem with excessive and inappropriate antibiotic use representing an important driver of this issue. Strategies to improve antibiotic prescription and dispensing are required in primary health care settings. The main purpose of this review is to identify and synthesize available evidence on the economic impact of educational interventions to reduce prescription and dispensing of antibiotics among primary health care professionals. Information about the clinical impact resulting from the implementation of interventions was also gathered. PubMed, Scopus, Web of Science and EMBASE were the scientific databases used to search and identify relevant studies. Of the thirty-three selected articles, most consisted of a simple intervention, such as a guideline implementation, while the others involved multifaceted interventions, and differed regarding study populations, designs and settings. Main findings were grouped either into clinical or cost outcomes. Twenty of the thirty-three articles included studies reporting a reduction in outcome costs, namely in antibiotic cost and associated prescription costs, in part due to an overall improvement in the appropriateness of antibiotic use. The findings of this study show that the implementation of educational interventions is a cost-effective strategy to reduce antibiotic prescription and dispensing among primary healthcare providers.

## 1. Introduction

Antibiotic resistance is globally recognized as a serious hazard to global public health [1], associated with negative impacts on health outcomes and expenditure [1]. The major driver of antimicrobial resistance has been a huge increase in antibiotic use, which increased by about 91% worldwide, and by 165% in low- and middle-income countries over the last decades [2,3]. Estimates show that drug-resistant infections will continue to rise dramatically, and by 2050 it is expected that 10 million deaths will occur each year and incur economic losses of over USD 100 trillion unless adequate interventions to limit unnecessary antibiotic use are implemented [1,4].

The prescription and dispensing of most antibiotics occur in primary healthcare facilities [5,6] which positions the health professionals as crucial stakeholders and partners in antimicrobial stewardship efforts [7]. Additionally, 25 to 50% of all antibiotics prescribed in primary healthcare are proved to be unnecessary, with substantial geographical and prescriber variability [8,9,10].

Several educational interventions have been conducted to improve or reduce antibiotic use [11,12,13], such as the distribution of educational materials (printed, electronic, or audio–visual materials that target the healthcare professional) [14,15,16,17], educational meetings (conferences, lectures, training courses, or workshops) [14,15,16,18,19,20,21], educational outreach visits (healthcare professionals receiving information from a trained professional in their practice setting) [16], audit and feedback (any summary of clinical performance of healthcare over a specified time period provided to the healthcare professional) [14,15,16,18,19,20,21,22,23], reminders (verbal, written, or electronic information intended to prompt a healthcare professional to recall information, including decision-support systems) [16,17,19,23,24], point-of-care tests (equipment to provide rapid diagnostic information to help reduce the uncertainty associated with clinical diagnosis) [15,18,20,21,25,26,27], communication strategies (any resource targeted at the healthcare professional that encourages discussion with a patient on management options) [14,15,20,21,28,29], mass media campaigns and delayed prescription strategy (i.e., any resource targeted at the healthcare professional that encourages authorization of a prescription for the patient to collect or use later than the initial consultation if symptoms do not improve) [16,19,23,24,25,30].

However, summaries of evidence on the most effective strategies considering their economic impact in primary healthcare are still lacking in the literature. A proper cost analysis of the existent educational interventions on antibiotic use in primary healthcare might provide insight into both the effectiveness and cost outcomes of antibiotic use, namely direct (medical and non-medical) and indirect (productivity loss, hospitalizations, etc.) costs, with the potential to be used by health professionals and policymakers [1,31]. Moreover, this information can help to support investments in appropriate, less expensive, and more beneficial strategies to reduce antibiotic use and, consequently, restrict the growing antibiotic resistance [1,31].

Thus, this study aimed to identify and synthesize available evidence on the economic impact of educational interventions to reduce the prescription and dispensing of antibiotics among physicians and pharmacists in primary healthcare. Furthermore, this review also gathered information about the clinical impact of educational interventions on the use, prescription and dispensing of antibiotics in primary healthcare settings.

## 2. Materials and Methods

### 2.1. Data Sources and Search Strategy

Searches in the International Prospective Register of Systematic Reviews (PROSPERO) and PubMed were conducted preceding the design of the present systematic review to exclude the existence of reviews or protocols with the same purpose as that presented in this review. No similar studies were found, and the review protocol was registered and is available at PROSPERO (CRD42022311272) [32]. This systematic review was performed in accordance with the Preferred Reporting Items for Systematic Review and Meta-analysis (PRISMA) (the PRISMA checklist can be found in Table S1, Supplementary Material) [33]. A systematic literature search was performed by a researcher on 21 February 2022, on the following electronic databases: PubMed, Scopus, Web of Science and EMBASE. Monthly automatic updates from each database were activated to ensure the update of the evidence. Search terms were based on a combination of keywords and MeSH terms on the review topic adapted for each database: (Intervention OR education* OR program OR “health promotion” OR session OR workshop) AND (antibiotic* OR antimicrobial*) AND (economic* OR cost* OR spending OR expend*) AND (“primary care” OR “primary health care” OR “Community Health Services” OR “general practitioner” OR practitioner OR “Community Pharmacy Services” OR “Pharmaceutical services”). Full search expression for each database is available in Text S1 (Supplementary Material).

The search was limited to terms found in titles, abstracts, and keywords. Reference lists of the selected articles were also scanned for other potentially eligible studies. Authors were contacted to obtain full texts when needed.

### 2.2. Study Selection and Eligibility Criteria

The screening process occurred in three steps and was conducted independently by two researchers (VR and ME): First, articles were excluded based on title, abstract and keywords. In step 2, full texts of the articles were evaluated to determine eligibility based on previously defined criteria. Then, in step 3, the selected articles were re-evaluated to assess their adequacy for data extraction. During the whole screening process, the researchers consecutively applied the following eligibility criteria:

Studies were included if they: (i) were written in English, Portuguese, French, or Spanish; (ii) were experimental, quasi-experimental or observational studies; (iii) described original educational interventions such as communication and education activities, stewardship programs, treatment algorithms, delayed treatment, peer or community oversight, medication reviews, or any other framework on antibiotic use in primary healthcare (strategies that were merely administrative, applied incentives or coercion were excluded from this study); (iv) included as target population physicians (general practitioners and all other specialists) and/or pharmacists; (v) described at least one economic effect measure of the educational interventions on the prescription behavior of physicians and/or dispensing behavior of pharmacists in primary healthcare facilities. Studies not conducted in humans (e.g., in vitro or animal studies), review articles, qualitative studies, magazines, news, research protocols, thesis, reports, dissertations, abstracts, communications, posters, letters to the editor, unpublished work, editorials, commentaries, books, book chapters without original data, guidelines, statements, position papers and case studies were excluded.

### 2.3. Data Extraction and Quality Assessment

Data extraction retrieved information on authors, year, country, study design, study population, disease, type of intervention, a brief description of the implemented educational interventions, antibiotic-related outcomes (cost-related and non-cost related) measured, study time period translated in months and the perspective assessed.

The risk of bias in each study was assessed independently by two researchers (VR and VN) using the Joanna Briggs Institute (JBI) critical appraisal checklist for economic evaluations [34,35], since this scale might be applied to studies including economic impact measures independently of their study design. Disagreements were resolved by consulting a third author (ME). Additionally, the inter-rater agreement of the quality assessment performed by the two reviewers was evaluated using Cohen’s kappa [36].

### 2.4. Data Syntheses and Analysis

The primary outcome was antibiotic cost. The results of the studies were summarized qualitatively and quantitatively. The decision matrix for the economic effects of the educational interventions had three possible outcomes adapted from a previous study [37]: (i) reject intervention: the educational intervention resulted in higher antibiotic cost, or had a higher cost and similar effectiveness, or similar costs and lower effectiveness, or higher cost and lower effectiveness; (ii) unclear: neither increase nor decrease in antibiotic costs were reported, or the intervention had lower cost and lower effectiveness, similar cost and similar effectiveness, or higher cost and higher effectiveness; (iii) favor intervention: the educational intervention resulted in savings in antibiotic cost, or had lower cost and similar effectiveness, similar costs and higher effectiveness, or lower cost and higher effectiveness.

## 3. Results

### 3.1. Study Selection and Quality Assessment

**Figure 1:**
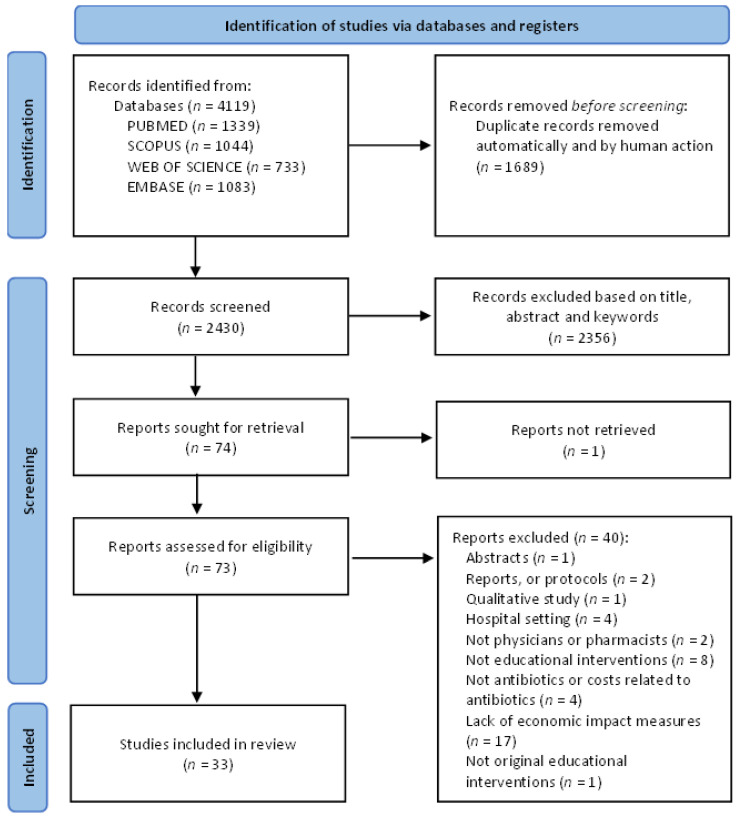
Decision Preferred Reporting Items for Systematic Review and Meta-analysis (PRISMA) flow diagram of the literature search (adapted from Page et al., 2021 [33]).

Figure 1 presents the literature search flow diagram. The systematic database search identified 4119 publications. After removing 1689 duplicates, the title, abstract and keywords were screened for 2430 papers. From these, 74 papers were full text screened. A total of 33 studies were included. Results of the quality assessment showed that only seven papers [38,39,40,41,42,43,44] presented more than two quality criteria classified as compliant and/or clear (Table S2 of Supplementary Material). The agreement between the two reviewers was substantial (k = 0.61, 95% CI 0.49–0.74, *p* < 0.001) and a final consensus was reached.

### 3.2. Study Characteristics

The characteristics of the 33 included studies are shown in Table S3 (Supplementary Material). Studies were published between 1996 [38] and 2021 [2,45], and most were conducted in the USA [38,39,40,46,47,48,49,50] and the Netherlands [51,52,53,54], and were randomized controlled trials [2,41,48,51,52,53,54,55,56,57,58,59,60,61,62,63] or pre–post studies [38,39,42,46,50,64,65]. All studies included primary care physicians or general practitioners (GPs), except for the study by Walker and colleagues that also included pharmacists [50]. Sample sizes ranged between 2 [38] and 3673 [57] participants. The most common diseases were respiratory tract infections (RTIs) [39,51,53,55,56,61,62,63,66], and either upper-RTIs (URTI) [39,55,61,62,63] or lower-RTIs (LRTIs) [51]. Most studies included patients from all age groups [2,39,43,44,45,48,50,56,57,58,59,60,65,66,67,68], only five included adults [47,49,51,52,55], four children [53,61,62,63] and two older adults [38,40,41,42,46,54,64,69].

### 3.3. Educational Intervention Types

The most common educational interventions were treatment guideline implementation [39,43,46,52,61,63] or guideline-based educational activities [41,58,59,62,65]. Some studies also investigated the effects of academic detailing [49,50,57,60,64,67], individualized prescription feedback [2,38,45,47,60,67], and training in enhanced communication skills [51,53,54]. The educational interventions implementation period ranged from 14 days [53] to 8 years [45]. Some studies compared similar periods of different years [2,38,39,40,42,43,44,49,52,53,59,60,64,65,66,67,68,69], within the same year [63] or both situations [41,50,57,62]. Only eleven studies [39,42,52,53,56,57,58,59,62,66,69] performed a follow-up analysis. While usual general practice care was used as a comparator in sixteen studies [2,38,41,47,52,53,55,56,57,58,59,61,62,63,64,65], the implementation of two or more distinct educational interventions in different study arms was also established as comparator in the articles of Michaelidis et al. and Naughton et al. [48,60].

### 3.4. Antibiotic Consumption and Appropriateness of Prescription

The appropriateness of antibiotic prescription and consumption was quantitatively evaluated in 15 of the included studies [2,39,42,43,44,45,46,49,50,52,57,62,65,67,68]. From these studies, only two [42,65] reported a decline in appropriate consumption and prescription of antibiotics and non-antibiotic therapeutic drugs, and five [2,43,46,50,67] stated no statistically significant change.

Three of the reviewed studies [52,57,62] specified that the improvement in appropriate behavior emerges from a reduction of broad-spectrum antibiotics use. Furst et al. [42], who assessed antibiotic consumption between 1999 and 2012, reported a significantly decreased consumption of antibiotics, except for β-lactamase-resistant penicillins [42] and also in restricted and non-restricted antibiotics consumption. Chazan and colleagues [44] showed a reduction in general antibiotic consumption, more specifically broad-spectrum antibiotic use in comparison with narrow-spectrum antibiotics. Concordantly, two additional studies [68] reported a significant decrease in broad-spectrum antibiotics along with an increase or stable use of narrow-spectrum antibiotics.

Armstrong et al. [46], who exclusively recruited bladder and kidney infection patients, revealed an increase in the administration of first-line trimethoprim-sulfamethoxazole or nitrofurantoin as initial antibiotic therapy accompanied by a decreasing consumption of fluoroquinolones [52].

Finally, Wei et al. [62] stated that to avoid patient complaints and the difficulty in differentiating between viral and bacterial infections, doctors tended to prescribe antibiotics even when not supported by the proper guidelines [65,68].

#### 3.4.1. Antibiotic Prescription Rate

A total of 26 studies [2,38,40,43,45,47,48,49,50,51,52,53,54,55,56,57,58,59,60,61,62,63,64,65,66,67] evaluated the impact of the implementation of educational interventions in improving the antibiotic prescription rate (APR) across primary healthcare providers. From these, 18 studies [2,40,45,47,48,49,51,52,54,55,56,57,58,59,61,62,63,66] showed statistically significant improvements regarding this outcome and 8 [38,43,50,60,64,65,67] also referred positive results, although not statistically significant for all the studied variables.

Specifically, the study of Aksoy and colleagues [45] conducted in Turkey over a period of eight years including family physicians reported decreased overall APR, mainly explained by a reduction in the number of boxes, items, and costs of antibiotic prescriptions. Another study [2] revealed that primary healthcare physicians who received a letter alerting the detection of their high prescription trends, tended to prescribe shorter-term prescriptions. Butler and colleagues [56] showed a significant reduction in terms of oral antibiotic dispensing, specifically for macrolides and phenoxymethylpenicillins (penicillin V), for all age groups and health conditions after implementation of the Stemming the Tide of Antibiotic Resistance (STAR) educational program when compared to the usual care. Two additional studies referred a reduction in prescription rates in specific subset of antibiotics, such as first-line antibiotics [64] and in broad-spectrum antibiotics [62].

Studies by Cals and colleagues [51] and Oppong et al. [54] reported that the reduction in APR was more pronounced among GPs that received an intervention combining the use of C-reactive protein (CRP) tests and training aimed at enhancing communication skills.

#### 3.4.2. Costs of Antibiotic Dispensing and Prescription

Antibiotic cost was the primary outcome of this study. The costs of antibiotic prescription and dispensing were quantitatively assessed in 29 [2,38,39,40,41,42,43,44,45,48,49,50,52,53,54,55,56,57,58,59,61,62,63,64,65,66,67,68,69] out of the 33 included articles. From these, only three studies [40,50,65] reported a significant increase in costs, while 26 showed a reduction in antibiotic cost.

Walker and colleagues [50] stated that the increasing utilization of generic first-line antibiotics was the main cause behind the reduction in cost per claim of antibiotics. Likewise, the study of March-López et al. [68] reported that the total spending on antibiotics showed a significant reduction, after the implementation of a multifaceted antimicrobial stewardship intervention, principally due to reductions in total spending of quinolones and amoxicillin/clavulanic acid.

Within the twenty-nine articles that analyzed antibiotic costs, five of them focused their cost analysis on the route of antibiotic administration, including oral [42,44,56,64,69] or, simultaneously, oral and intravenous [69] antibiotics.

#### 3.4.3. Antibiotic Treatment Success and Quality-Adjusted Life Years

The success of treatment using antibiotics was evaluated in five studies [39,46,49,52,68], with only one study [39] reporting positive and significant results.

While the study by Armstrong and colleagues [46] focused their analysis only on bladder and kidney infections, three other studies analyzed a wider set of pathological conditions, namely urinary tract infections [68], pharyngotonsillitis [68], and acute respiratory infections [39,49,68]. Armstrong et al. [46] demonstrated that the overall treatment success rate of kidney and bladder infections remained essentially unchanged; however, after the treatment guideline implementation, the highest success rate results were achieved using antibiotics such as fluoroquinolone and doxycycline groups, respectively. Another study [39] showed that only 1% of the attending patients with URTI progressed to pneumonia.

Moreover, ten studies [39,46,47,48,49,51,52,53,54,56] also addressed the number of hospitalizations and/or re-consultations, the latest including situations of physician home visits and telephone consultations.

Quality-adjusted life-years (QALYs) were measured in two studies [47,54]. Gong et al. [47] reported that the three intervention arms (i.e., suggested alternatives; accountable justifications; and peer comparison) yielded more QALYs at a lower cost when compared with the control group.

### 3.5. Economic Effects of the Educational Interventions

The implementation of educational interventions was associated with savings in antibiotic cost, or lower cost in 30 of the 33 included studies.

In 22 of the 33 included papers, a cost-analysis (CA) was performed [2,38,39,40,41,42,43,44,45,49,50,52,55,56,57,59,64,65,66,67,68,69], 10 studies performed a cost-effective analysis (CEA) [47,48,51,53,54,58,60,61,62,63] and Armstrong and colleagues conducted a cost-minimization analysis (CMA) in their study [46] (Table 1). Most of them assessed data from a health service perspective [2,39,40,41,42,43,44,45,50,51,54,55,56,57,58,59,61,62,63,65,66,67,68,69], three from a societal perspective [47,48,53] and two studies from a managed care organization perspective [46,64].

All costs mentioned across the included articles were acquired retrospectively, except in two studies [48,53] that obtained costs from the literature and three other studies [47,49,54] that collected this data.

The main direct medical costs assessed in the included studies were expenses associated with healthcare providers (clinician salaries, out-of-hours care costs, telephone calls), hospitalization (e.g., physician consultations, laboratory tests and material charges) and drugs (e.g., treatment, medication, and prescription expenditures). The costs involved in the intervention design and implementation (organizing and administrative intervention costs, seminars/workshops/online training courses costs, educational materials costs, travel costs, outreach visits costs, and staff costs) were also reported in several studies [38,43,47,48,49,51,52,53,54,56,57,58,60,64,69]. The study of Gillespie and colleagues [58] also highlighted some additional costs related to electronic software needed to support interventions.

A total of 13 different modelling techniques were applied in 16 of the included studies, namely an econometric model [46], a covariance model [56], a linear [52], logistic [52,63], and time series-regression model [46], a hierarchical model [54,59], a linear-mixed [57,67], and function model [42], a generalized estimating equations model [58], a thirty-year Markov model [47], a cost-effectiveness model [48,60], a generalized linear model [62] and a multivariate model [65]. Seven studies [47,48,51,53,54,58,63] employed incremental cost-effectiveness ratio (ICER) to assess the outcome. Net monetary benefit (NMB) [47,48,51], willingness-to-pay (WTP) [47,48,51,54,58] and return on investment [57] were other measurements used to assess the cost outcomes.

## 4. Discussion

This study systematically reviewed the economic impact of educational interventions implemented to significantly improve or reduce antibiotic prescription and dispensing among physicians and pharmacists in primary healthcare settings.

### 4.1. Antibiotic Consumption and Prescription

Overall, educational interventions were demonstrated to have a positive impact in both the conscientious consumption of antibiotics and appropriateness of antibiotic prescription and dispensing in primary healthcare provider prescriptions. Evidence from the thirty-three included articles showed that one of the major aims of educational interventions is to guide antibiotic prescription, which predominantly culminate in favorable outcomes, either by decreasing the overall amount of antibiotic prescriptions or by improving prescription quality. This positive impact on the appropriateness of antibiotic prescriptions, in primary healthcare settings, is in accordance with the existing literature [70,71]. The appropriate antibiotic prescription was associated with short- and long-term cost reduction, since it promoted cost reductions associated with less hospitalizations, second-line inpatient antibiotic use and non-antibiotic drug costs (i.e., equipment, workload, etc.), for instance, resulting in improvements in morbidity and mortality [57].

Nevertheless, the inappropriate use and prescription of antibiotics are not always easy to reverse since, in many cases both clinicians’ and patients’ attitudes may present barriers to the implementation of good practices. The absence of patient awareness on the hazards caused by antibiotic-resistance, lack and/or ineffective communication between prescribers, patients and pharmacists, the pressure by patients towards physicians to prescribe antibiotics, the clinicians’ fear of patients worsening, and the expectations of practitioners and patients may represent barriers to provision of the correct prescription and dispensing of antibiotics [2,60,62,70,72]. The study of Wei et al. [62] reported examples of reasons provided by doctors to maintain their antibiotic prescription trends, namely difficulty in differentiating between viral and bacterial infections and to avoid patient complaints.

Generally, antibiotic use is higher in more deprived areas, explained by factors such as the lack of regulations to prevent the over-the-counter sale of antibiotics, the limited availability of essential diagnostic procedures, the inadequate training of healthcare professionals, and the high burden of illness and comorbidities [73]. However, the literature also showed extremely high values of antibiotic prescription and dispensing in high-income countries, namely in primary healthcare facilities where most antibiotics are supplied [72]. In the study of Aksoy and colleagues [45], a reduction or maintenance in antibiotic prescription behavior was described; however, the overall drug prescription raised significantly. In comparative terms, it would be interestingly to verify this trend (i.e., reduction in antibiotic prescription and simultaneous increase in prescription of the overall drugs), in the other included studies; however, this analysis was not possible since none of the other included studies evaluated the same outcomes.

As mentioned before, the antibiotic prescription quality showed to be a crucial factor in the improvement of antibiotic prescription practice. However, prescribers tended to favor long-term prescriptions, therapies combining multiple antibiotics with similar pharmacological characteristics, as well as prescription of broad-spectrum antibiotics, all factors negatively associated with poor prescription quality, and, indirectly, with antibiotic-resistance [6,74]. Three studies [52,57,62] out of the included papers stressed the importance of reducing the use of broad-spectrum antibiotics for improving quality of antibiotic prescription. Concordantly, two additional studies [44,68] reported a significant decrease in the use of broad-spectrum antibiotics in favor of narrow-spectrum antibiotics, which [43,44,68] showed to be an appropriate alternative [43,44,68], since therapy with narrow-spectrum antibiotics is associated with a lower risk of drug-related adverse effects and a higher health-related quality of life [74,75]. Other concerns associated with antibiotic misuse were prescription without clinical indication and improper consumption associated with the choice of a suitable molecule and dosage of therapy according to patients’ characteristics and location of infection [73]. To sum up, despite the efforts to increase appropriate prescription attitudes among primary physicians using educational interventions, several studies demonstrated that additional changes/interventions are still required since prescription values remain above the desirable goals [38,42,43,50,53,60,64,65,67].

### 4.2. Antibiotic Cost and Costs of Antibiotic Prescription

The cost of the prescribed medicines is one of the most important drug utilization indicators allowing the assessment of rational use of drug performance by clinicians [76]. Within the scope of this review, two of the major outcomes analyzed in twenty-nine [2,38,39,40,41,42,43,44,45,48,49,50,52,53,54,55,56,57,58,59,61,62,63,64,65,66,67,68,69] of the included studies were the changes in antibiotic cost and costs of antibiotic prescription and dispensing. To reduce antibiotic costs and associated costs, one of the strategies currently adopted is the availability of generic first-line antibiotics that resulted in significant cost savings over their branded and broader-spectrum counterparts [62]. This finding is also supported by the study of March-Lopez et al. [68] who reported a significant reduction in the total spending on antibiotics due to reductions in total spending on broad-spectrum antibiotics, and by Walker and colleagues [50] who stated that the reduction in the cost per claim of antibiotics was mainly triggered by the increasing utilization of generic first-line antibiotics. This is in line with previous literature reporting that the use of broad-spectrum antibiotics, which are commonly more expensive, and alteration in clinicians’ prescription practices, namely the prescription of second-line antibiotics, are considered some of the main drivers behind extra costs in healthcare [77].

In addition to monetary effects, directly related to antibiotics and their associated costs, inappropriate antibiotic prescriptions and dispensing also have negative effects on productivity [77,78]. Productivity losses are related to the amount of work time lost, due to lack and/or non-productivity as a result of reduced concentration, as well as the expected number of additional hours needed to conclude the regular amount of work [53,77]. The studies of Calls et al. [51] and Dekker et al. [53] were unique in that they considered productivity loss, with Dekker and colleagues reporting that the productivity loss of parents represented the highest costs regarding the intervention group, whereas Calls et al. did not observe any significant difference. These societal benefits of interventions are crucial and should thus not be underestimated. Nevertheless, only three studies [47,48,53] assessed data from a societal perspective.

### 4.3. Educational Interventions

Treatment guideline implementation [39,43,46,52,61,63] and guideline-based educational activities [58,59,62,65] were the most implemented interventions throughout the included studies. A systematic review regarding the impact of guideline adherence regarding antibiotic prescription stated that, in general educational interventions focused on adherence to guidelines are enough to improve the quantity and quality of prescriptions [79]. However, the implementation of more complex interventions, i.e., multifaceted interventions, comprehending individualized prescription feedback [2,38,45,47,60,67], academic detailing [49,50,57,60,64,68] and training in enhanced communication skills [51,53,54] were also often applied in primary healthcare settings. Communication skills training and strategies to communicate clinical information were also adopted strategies to persuade clinicians to change their practice performance [43,51,53,54,56,62].

### 4.4. Economic Effects of the Educational Interventions

Educational interventions showed to be of most relevance, especially for policymakers, regarding enhancement of prescription and dispensing, among physicians and pharmacists in primary healthcare settings, and for promoting cost savings [57].

Despite being stated in the literature that CEA is one of the most reliable tools of process and economic analysis [80], more than half of the included studies [2,38,39,40,41,42,43,44,45,49,50,52,55,56,57,59,64,65,66,67,68,69] implemented a CA. CEA is an extremely useful method in terms of direct comparison of different interventions with identical outcomes [80,81]. When compared to other methods of analysis it presents the benefit of bringing into focus the relative advantages and disadvantages of implementing several interventions from both cost and clinical perspectives, since it allows, simultaneously, the identification of the intervention that confers more benefits to patients, and that provides more cost savings for health systems to help inform policy decisions [81].

As seen within the papers that performed a CEA, there is ample variation in the reported outcomes measured (ICER, WTP, NMB) throughout the included economic evaluation studies, that in conjunction with other methodological discrepancies are responsible for impeding, in a certain mode, the utility of data on the effectiveness and costs in the ongoing practice in primary healthcare facilities. Return on investment, another measure of cost outcome and a method of cost–benefit analysis, allows the measurement of educational intervention costs and the financial recovery of these interventions estimated as a net benefit, i.e., the total benefit minus the total cost, over total cost [82]. Figueiras et al.’s study [57] was the only that reported the return on investment as a measure of cost outcomes. Nevertheless, this type of economic analysis frequently neglects the patients’ health, since it is based on temporary recovery. Thus, to surpass this limitation, it is crucial to adopt other outcome measures, that consider the quality of life in patients who experienced the clinical outcomes, enabling comparisons between economic evaluations [82]. QALY is an example of one of those effectiveness-standardized outcome measures that were implemented by Oppong et al. [54] and Gong et al. [47].

Resistance patterns, which may diverge according to the geographical regions, and the financial difficulties of healthcare systems represent huge challenges to the implementation of cost-effectiveness interventions among countries and to apply knowledge translation to current clinical practice [82]. Furthermore, evidence for the cost-effectiveness of implementing educational interventions still requires further exploration since although numerous studies [39,42,52,53,56,57,58,59,62,66,69] have been performed with the application of a follow-up analysis to check the middle- and long-term effects of the intervention, the evidence is not yet consistent on this topic.

Cost-effectiveness presents an example of an economic health evaluation that is crucial for policymakers and healthcare providers to formulate the best decisions regarding alternative methods of action, since in addition to accessing health benefits, i.e., effectiveness, it also allows the evaluation of relevant information about the costs needed to implement educational interventions [37]. Considering the three possible outcomes, favor intervention, unclear and reject intervention, previously identified in the study of Munn et al. [37], three studies [40,41,61] did not report favoring of interventions. Possible explanations for this finding were the selection of unsuitable control arms (e.g., historical controls), the reduced impact, as less than expected of the implemented interventions, the inadequate practice settings where interventions were implemented and the lack of sensitive prescribing habits regarding cost information.

The findings of this study did not allow us to identify the most cost-effective strategy for improving or reducing antibiotic prescription and dispensing in primary healthcare. However, overall, the implemented educational interventions seemed to result in significantly positive cost and clinical outcomes via the adoption of appropriate antibiotic prescription and dispensing practices in primary healthcare settings.

### 4.5. Strengths and Limitations

This systematic review was associated with some limitations that merit discussion.

Firstly, publication bias might have affected our findings. As previously stated, four scientific databases (PubMed, Scopus, Web of Science, EMBASE) were selected for searching potential selection of studies. Thus, grey literature, that includes a wide range of documents difficult to search and retrieve, such as studies with null findings, abstracts, and other unpublished documents were not included. However, the defined inclusion criteria allowed the coverage of a wide range of studies with different settings and designs.

Secondly, our findings were limited by the level of heterogeneity within the included studies, observed in the different study designs, data collection methods, statistical cost analysis methodologies and ways of presenting cost values. To overcome this limitation, we transformed all costs into Purchasing Power Parities (ppp) and this allowed us to equalize the purchasing power of different currencies, by eliminating the differences in price levels across countries. The high heterogeneity also hindered us from drawing closer comparisons between costs and performing a meta-analysis. Additionally, some common issues observed across the analyzed papers were the lack of randomization of the study population and/or the absence of a control group, resulting in a reduced ability to determine whether the results were due to the educational intervention or external factors. The imbalance between participants, i.e., more primary care physicians than pharmacists, may have also limited generalization of results. Another limitation is associated with the timeline of some of these studies, since twenty-two of the revised papers, did not perform or report follow-up results, so it is not possible to conclude whether the interventions were effective over middle- and long-term periods. Finally, most of the included studies did not report sociodemographic characteristics of the physicians/pharmacists and the few [2,40,41,44,52,55,56,57,59,61,62,63,67] that reported did not explore correlations with the trends of antibiotic prescription, dispensing and cost.

Despite these limitations, we may highlight the extensive, rigorous, and systematic search across the four distinct databases (PubMed, Scopus, Web of Science, EMBASE) following well-established and updated guidelines for conducting systematic reviews [33,83], which might be considered the main strength of this study.

## 5. Conclusions

Our results suggest that educational interventions were associated with improvements in the overall prescription rate, dispensing, and consumption of antibiotics as well as significant reduction in antibiotic costs. These results support the need for public health actions to qualify primary healthcare providers, principally in low- and middle-income countries, through the implementation of cost-effective educational interventions to reduce the prescription and dispensing of antibiotics in primary healthcare settings and a consequent decrease in the associated healthcare costs.

## Figures and Tables

**Table 1:** Summary on the economic effects of the educational interventions.

Author Year, Country	Antibiotic-Related Measures (Not Cost-Related)	Type of Economic Evaluation	Antibiotics Cost before Intervention or in the CG	Antibiotics Cost after Intervention or in the IG	Cost of the Intervention(s)	Incremental Cost-Effectiveness (Change in Costs)	Interpretation
Aksoy et al., 2021 [45] Turkey	-Reduction in antibiotics prescriptions from 34.94 to 30.25% -Reduction in antibiotics items from 14.14 to 4.12% -Reduction in antibiotics boxes from 12.71 to 6.64%	Cost analysis	-Antibiotics cost before intervention: 11.38%	-Antibiotics cost after intervention: 4.12%	NR	Costs savings of 7.26%	Favor intervention
Armstrong 2001 [46] USA	-Kidney infection: 49% success rate with the antibiotics treatment guideline compared to 42% with no guideline (*p* = 0.59) -Bladder infection: 72% success rate with the antibiotics treatment guideline compared to 42% with no guideline (*p* = 0.68)	Cost minimization analysis	Before intervention: Kidney infection event cost: 452 ± USD 1287~452 ± 1287 ppp Bladder infection event cost: 125 ± USD 611~125 ± 611 ppp	After intervention: Kidney infection event cost: 289 ± USD 470~289 ± 470 ppp Bladder infection event cost: 116 ± USD 400~116 ± 400 ppp	NR	-Decrease of 36% in health event costs (*p* = 0.696) -Decrease of 7% in health event costs (*p* < 0.05)	Favor intervention
Balcioğlu et al., 2017 [55] Turkey	APR: IG (Algorithm group): *n* = 23 (0.1%) CG: *n* = 65 (0.4%)	Cost analysis	Prescription cost in CG: TRY 26.9~19.44 ppp	Prescription cost in IG: TRY 15.4~11.13 ppp	NR	Cost savings of 8.31 ppp	Favor intervention
Butler et al., 2012 [56] UK	-Reduction of 4.2% (95% confidence interval 0.6% to 7.7%) in total oral antibiotic dispensing per 1000 patients -No significant differences in hospital admissions and re-consultation rates between IG and CG	Cost analysis	Antibiotics costs in baseline: CG: GBP 2254.6~3211.68 ppp IG: GBP 2199.7~3133.48 ppp	Antibiotics costs in follow-up CG: GBP 2252.3~3208.40 ppp IG: GBP 2078.9~2961.40 ppp	Total costs of intervention: GBP 96,460~137,407.41 ppp	-Cost savings of GBP 120.8~172.08 ppp in the intervention group -Decrease of 5.5% (−0.4; 11.4, *p* = 0.07) in antibiotic cost	Favor intervention
Cals et al., 2011 [51] Netherlands	-IG1 (CRP group): 43 ± 39.1 antibiotics at index consultation; 3.35 ± 4.54 days of work; EUR 98 ± 89.1 diary cost -IG2 (Communication skills training group): 28 ± 33.3 antibiotics at index consultation; 3.37 ± 4.02 days of work; EUR 74 ± 88.1 diary cost -IG3 (CRP + communication skills training group): 27 ± 23.1 antibiotics at index consultation; 3.39 ± 4.08 days of work; EUR 110 ± 94 diary cost -CG (Usual care): 80 ± 66.71 antibiotics at index consultation; 3.37 ± 3.77 days of work; EUR 104 ± 86.7 diary cost	Cost effectiveness analysis	NR	NR	Total costs -IG1: EUR 37.58 ± 45.24~117.22 ± 54.11 ppp -IG2: EUR 25.61 ± 44.49~30.63 ± 53.22 ppp -IG3: EUR 37.78 ± 42.08~45.19 ± 50.33 ppp -CG: EUR 35.96 ± 58.12~43.01 ppp	Incremental cost-effectiveness ratio: -IG1: 5.79 -IG2: Dominant -IG3: 4.15	Favor intervention
Chazan et al., 2007 [44] Israel	Seasonal intervention: -change from 27.8 to 23.2 DDD/1000 patients/day in total antibiotics use -NS difference in the narrow-spectrum antibiotics use Continuous intervention: -change from 28.7 to 22.9 DDD/1000 patients/day in total antibiotics use -reduction in broad-spectrum antibiotic use (17.6%)	Cost analysis	NR	NR	NR	-Cost savings of USD 186~186 ppp per 1000/patients/season in the seasonal intervention -Cost savings of USD 330~330 ppp per 1000/patients/season in the continuous intervention	Favor intervention
Coenen et al., 2004 [52] Netherlands	In IG: -APR, pre-test: 43%; post-test: 27.4%; −15.6% of change; -Use of recommended antibiotics, pre-test: 40.1%; post-test: 53.8%; −13.6% of change In CG: -APR, pre-test: 37.8%; post-test: 28.7%; −9.1% of change; -Use of recommended antibiotics, pre-test: 37.5%; post-test: 37.4%; −0.1% of change	Cost analysis	Medication cost in CG, pre-test: EUR 21.48~23.66 ppp post-test: EUR 22.35~24.61 ppp	Medication cost in IG, pre-test: EUR 22.35~24.61 ppp post-test: EUR 16.75~18.75 ppp	NR	Change in medication cost: CG: EUR 0.87~0.96 ppp IG: EUR 6.11~6.73 ppp Mean difference: −6.76 (95% CI: −12.30; −1.89)	Favor intervention
Conklin et al., 2009 [64] Pennsylvania	In kiosk prescribers: First line APR decreased from 49.1 to 47.0%; median decrease of 2.3% (SD, 13.0%)	Cost analysis	In non-kiosk prescribers: Mean antibiotic cost per claim: USD 33.56~33.56 ppp	In kiosk prescribers: Mean antibiotic cost per claim: USD 29.42~29.42 ppp	NR	A median cost decrease of antibiotic per claim of USD 3.56~3.56 ppp	Favor intervention
Dekker et al., 2018 [53] Netherlands	IG: mean antibiotics of 0.25; 4.5 h of work absence; 0.5 h of non-productivity CG: mean antibiotics of 0.50; 3.1 h of work absence; 1.5 h of non-productivity	Cost effectiveness analysis	-Prescribed medication cost per child: EUR 8.81~11.31 ppp -Mean total cost: EUR 207.68 (140–284)~267.28 (18.18; 365.50) ppp	-Prescribed medication cost per child: EUR 4.77~6.14 ppp -Mean total cost: EUR 217.95 (150; 301)~280.50 (193.05; 470.40) ppp	Costs of intervention per child: EUR 2.9~3.73 ppp	Cost savings per child of EUR 4.04~5.20 ppp Mean incremental cost-effectiveness ratio: EUR 0.85~1.09 ppp per percentage decrease in antibiotic prescription	Favor intervention
Farris et al., 1996 [38] USA	1st study period -change in APR: −9.5% -change in amoxicillin ratio: −5.5% 2nd study period -change in APR: −3.2% -change in amoxicillin ratio: −12.1%	Cost analysis	NR	Average cost per prescription: 1st study period, USD 40.54~40.54 ppp 2nd study period, USD 41.08~41.08 ppp	USD 3700~3700 ppp	Cost savings: USD 3784~3784 ppp Net savings: USD 84~84 ppp	Favor intervention
Figueiras et al., 2020 [57] Spain	% of reduction in IG relative to CG: -Reduction of −4.23 (95% CI: −5.26; −3.21) DDD in antibiotics for systemic use; -Reduction of −6.51 (95% CI: −7.92; −5.22) DDD in penicillins; -Reduction of −3.89 (95% CI: −6.18; −1.65) DDD in cephalosporins; -Reduction of −3.45 (95% CI: −5.23; −1.70) DDD in macrolides, lincosamides and streptogramins -Reduction of −0.47 (95% CI: −2.37; 0.93) DDD in quinolones; Reduction of −8.97 (95% CI: −13.99; −4.12) in consumption ratio of broad-to narrow-spectrum antibiotics	Cost analysis	NR	NR	Total cost of intervention: 105. EUR 834~168.79 ppp	Savings in absolute direct costs of −4.33% (95% CI: −5.38; −3.29) Savings in cost per physician of −4.33% (95% CI: −5.38; −3.29) Savings in direct costs per 1000 inhabitants −4.46 (95% CI: −5.54; −3.4)% Total direct cost savings of EUR 697.38 (−861.79; −533.22)~−1112.25 (−1374.47; −850.43) ppp	Favor intervention
Furst et al., 2015 [42] Slovenia	Antibiotics prescriptions decreased 53%	Cost analysis	NR	NR	Cost of the intervention EUR 325,000~591,596.64 ppp + EUR 500,000~840,336.13 ppp	Cost savings in antibiotics of EUR 13.1 million~22.02 million ppp	Favor intervention
Gillespie et al., 2016 [58] Ireland	-IG1 (Arm A): 78.6% antimicrobial prescriptions; EUR 84.2 (SD: 24.6) of cost per consultation; 68.2% first-line antimicrobials -IG2 (Arm B): 75.8% antimicrobial prescriptions; EUR 88.7 (SD: 24.3) of cost per consultation; 66.5% first-line antimicrobials -CG: 66.5% prescriptions; EUR 67 (SD: 26.1) of cost per consultation; 44.1% first-line antimicrobials	Cost effectiveness analysis	Antimicrobial prescriptions cost per consultation in CG: EUR 5.3 (SD: 4.0)~6.68 (SD: 5.04) ppp	Antimicrobial prescriptions cost per consultation: -IG1: EUR 5.1 (SD: 3.4)~6.42 (SD: 4.28) ppp -IG2: EUR 5.2 (SD: 3.5)~6.55 (SD: 4.41) ppp	Cost related to intervention set-up, audit and feedback -IG1: EUR 16.3~20.53 ppp per consultation -IG2: EUR 16.4~20.65 ppp per consultation	ICERs per % increase in first-line antimicrobial prescription for UTI: -IG1: EUR 64.2 (95% CI: 22.0, 121.8)~80.86 (95% CI: 27.71; 153.40) ppp -IG2: EUR 105.4 (95% CI: 46.6, 241.7)~132.75 (95% CI: 58.69; 304.41) ppp	Favor intervention
Gong et al., 2019 [47] USA	-CG: 14.68 QALYs; Intervention cost of 178.21$~178.21 ppp -IG1 (suggested alternatives): 14.73 QALYs; Intervention cost of USD 173.22~173.22 ppp -IG2 (accountable justifications): 14.74 QALYs; Intervention cost of USD 172.82~172.82 ppp -IG3 (peer comparison): 14.74 QALYs; Intervention cost of USD 172.52~172.52 ppp	Cost effectiveness analysis	NR	NR	Cost of implementation-IG1: 1.91 (0–5.73) -IG2 3.82 (0–9.55) -IG3 0.95 (0–3.82)	Overall budget impact: -CG: USD17.82 million~17.82 million ppp -IG1: USD 17.32 million~17.32 million ppp -IG2: USD 17.28 million~17.28 million ppp -IG3: USD 17.25 million~17.25 million ppp	Favor intervention
Hux et al., 1999 [41] Canada	IG: pre, 67.2%; post, 69.8% in first-line antibiotics CG: pre, 68.5%; post, 66.8% in first-line antibiotics (*p* < 0.001)	Cost analysis	Median antibiotic cost in CG: pre, CAD 10.78~8.77 ppp; post, CAD 14.15~11.52	Median antibiotic cost in IG: pre, CAD 11.50~9.36 ppp; post, CAD 11.55~9.41 ppp	NR	No savings in median antibiotic cost in the IG and an increase in CG cost	Unclear
Lanbeck et al., 2016 [69] Sweden	IG: 7193 days at the hospital; 108 deaths; 180 patients readmitted within 28 days. CG: 7402 days at hospital; 117 deaths; 203 patients readmitted within 28 days.	Cost analysis	Oral antibiotic treatment cost: SEK 94,367~10,695.57 ppp Intravenous antibiotic treatment cost: SEK 690,440~78,254.56 ppp	Oral antibiotic treatment cost: SEK 46,850~5309.99 ppp Intravenous antibiotic treatment cost: SEK 616,264~69,847.44 ppp	Cost of intervention: SEK 161,990~18,359.97 ppp	Cost savings in oral antibiotic of SEK 47,517~5385.58 ppp Cost savings in intravenous antibiotic of SEK 74,176~8407.12 ppp	Favor intervention
Le Corvoisier et al., 2013 [59] France	IG: Reduction in antibiotics prescriptions from 15.2 ± 5.4% to 12.3 ± 5.8% (−2.8% [95% CI: −3.8; 1.9]; *p* < 0.001) CG: Increase in antibiotics prescriptions from 15.3 ± 6.0% to 16.4 ± 6.7% (+1.1% [95% CI; 0.4; 1.8], *p* < 0.01)	Cost analysis	Cost of antibiotic prescriptions in CG: EUR 393 (95% CI: 201; 585)~429.04 (95% CI: 219.43; 638.65) ppp	Cost of antibiotic prescriptions in IG: -EUR 313 (95% CI: −512; −113)~341.70 (95% CI: −558.95; −123.36) ppp	NR	Significant reduction (EUR 80~87.34 ppp) in antibiotic prescription cost (*p* < 0.001)	Favor intervention
Madridejos-Mora et al., 2004 [67] Spain	Antibiotics over prescription: -IG (individualised feedback group): Pre, 16.4 (SD: 7.27); Post, 16.4 (SD: 6.15); *p* = 0.986 -CG (minimal intervention group): Pre, 15.7 (SD: 8.44); Post, 13.7 (SD: 6.81); *p* = 0.006	Cost analysis	Antibiotics cost in CG: Pre, 3.18 (SD: 1.59) EUR/inhabitant~4.15 (SD: 2.08) ppp/inhabitant Post, 3.25(SD: 1.31) EUR/inhabitant~4.24 (SD: 1.71) ppp/inhabitant	Antibiotics cost in IG: Pre, 2.94 (SD: 1.89) EUR/inhabitant~3.84 (SD: 2.47) ppp/inhabitant Post, 2.49 (SD: 1.42) EUR/inhabitant~3.25 (SD: 1.85) ppp/inhabitant	NR	Significant reduction (EUR −0.45~0.59 ppp/inhabitant) in antibiotic prescription cost (*p* = 0.004)	Favor intervention
March-Lopez et al., 2020 [68] Spain	-A decrease from 26.99 to 22.41% (−4.57%; *p* < 0.05) in antibiotic consumption -An increase from 31.32 to 32.35% (+1.04%; *p* < 0.05) in narrow-spectrum antibiotics	Cost analysis	Total antibiotic spending in 2016 (control): EUR 905,700.76~1,444,498.82 ppp	Total antibiotic spending in 2018 (sustainability phase): EUR 793,765.89~1,265,974.31 ppp	NR	Cost savings in antibiotic spending: EUR 111,934.87~178,524.51 ppp	Favor intervention
McNulty et al., 2000 [43] UK	-IG (workshop group): a reduction of −2458 (−3.4%) in antibiotics units; a reduction of −139 (−0.9%) in narrow-spectrum antibiotics; a reduction of −1612 (−15.4%) in broad-spectrum antibiotics -CG (non-workshop group): a reduction of −1209 (−2.2%) in antibiotics units; a reduction of −1248 (−11.7%) in narrow-spectrum antibiotics; an increase of 561 (6.5%) in broad-spectrum antibiotics	Cost analysis	CG: an increase of GBP 8710~12,354.61 ppp (3.8%) in antibiotics units; a reduction of -GBP 1160~1645.39 ppp (−10.8%) in narrow- spectrum antibiotics; an increase of GBP 7100~10,070.92 ppp (8.8%) in broad-spectrum antibiotics	IG: a reduction of -GBP 3400~4822.70 ppp (−3.4%) in antibiotics units; an increase of GBP 220~312.06 ppp (1.5%) in narrow- spectrum antibiotics; a reduction of GBP 8330~11,815.60 ppp (−9.3%) in broad-spectrum antibiotics	NR	-Cost savings in antibiotic units of 3.8% (NS) -Cost savings in broad-spectrum antibiotics of −9.3% (*p* < 0.001) -Cost increase in narrow-spectrum antibiotics of 1.5% (*p* = 0.016)	Favor intervention
Me’emary et al., 2009 [66] Syria	-CG (baseline survey group): 86.5% of antibiotic prescriptions; SYP 356,223~NA ppp of total antibiotics cost -IG (impact survey group): 62.8% of antibiotic prescriptions; SYP 157,182~NA ppp of total antibiotics cost	Cost analysis	CG: 66.5% of total antibiotics cost	IG: 55.1% of total antibiotics cost	NR	Cost savings of −17.1% (*p* < 0.001) in antibiotics cost	Favor intervention
Michaelidis et al., 2015 [48] USA	-IG1 (printed decision support): 3.78 antibiotic prescriptions per 5 cases of disease; <1.9 days of work loss compared to CG; -IG2 (computerized decision support): 3.94 antibiotic prescriptions per 5 cases of disease -CG (usual care): 4.60 antibiotic prescriptions per 5 cases of disease	Cost effectiveness analysis	NR	NR	-IG1: USD 2574~2574 ppp -IG2: USD 2802~2802 ppp -CG: USD 2768~2768 ppp	The IG1 showed to be the most cost-effective strategy to reduce antibiotic use, specifically safely avoiding antibiotics prescriptions of −0.16 and −0.82 (incremental effectiveness) compared to IG2 and CG	Favor intervention
Naughton et al., 2008 [60] Ireland	Immediate response: -IG1 (postal bulletin group): decrease of −0.02 (−0.04; −0.001) in APR; increase of 0.02 (0.002; 0.05) in first-line antibiotics; decrease in second-line antibiotics of −0.03 (−0.05; −0.01) in co-amoxiclav and −0.02 (−0.03; −0.007) in cephalosporins -IG2 (academic detailing group): decrease of −0.02 (−0.03; −0.001) in APR; increase of 0.05 (0.01; 0.09) in first-line antibiotics; decrease in second-line antibiotics of −0.03 (−0.05; −0.01) in co-amoxiclav and −0.02 (−0.03; −0.003) in cephalosporins	Cost effectiveness analysis	NR	NR	Total cost of implementation in IG1 was EUR 210,000~222,457.61 ppp with a cost per GP practice of EUR 175~185.38 ppp Total cost of implementation in IG2 was EUR 1,868,000~1,978,814 ppp with a cost per GP practice of EUR 1556~1648.31 ppp	The cost-effectiveness ratio for the IG1 was EUR 88~93.22 ppp per %change in practice compared with EUR 778~824.15 ppp for academic detailing	Favor intervention
O’Connor et al., 1999 [39] USA	-Pre-guideline: 24% using antibiotics and 76% not using antibiotics -Post-guideline: 16% using antibiotics and 84% not using antibiotics	Cost analysis	Pre-guideline cost of initial care: USD 37.8~37.8 ppp	Post-guideline cost of initial care: USD 36.2~36.2 ppp	NR	Net savings of 4.2% (NS)	Favor intervention
Oppong et al., 2018 [54] Belgium, Netherlands, Poland, Spain, UK	-EG1 (CRP group): 222 (33.64%) antibiotic prescriptions; 0.0651 QALYs; -EG2 (Communication skills group): 303 (40.95%) antibiotic prescriptions; 0.0651 QALYs; -EG3 (CRP + communication skills group): 242 (34.13%) antibiotic prescriptions; 0.0648 QALYs -CG (Usual care): 307 (59.61%) antibiotic prescriptions; 0.065 QALYs;	Cost effectiveness analysis	Antibiotic cost: -CG: EUR 27.96~41.24 ppp	Antibiotic cost: -EG1: EUR 49.34~72.77 ppp -EG2: EUR 39.56~58.35 ppp -EG3: EUR 60.32~88.97 ppp	Cost of delivering the intervention: -EG1: EUR 11.42 (SD: 7.45)~16.84 (SD: 10.99) ppp -EG2: EUR 5.62 (SD: 3.69)~8.29 (SD: 5.44) ppp -EG3: EUR 13.43 (SD: 8.53)~19.81 (SD: 12.58) ppp	-EG3: ICER of EUR 338.89~499.84 ppp -EG1: ICER of EUR 176.53~260.37 ppp -EG2: ICER of EUR 68.80~101.47 ppp All per percentage reduction in antibiotic prescription when compared with CG	Favor intervention
Ornstein et al., 1999 [40] USA	CG (non-cost information in prescriptions): 23.85% of antibiotics prescriptions EG (cost information in prescriptions): 21.59% of antibiotics prescriptions *p* = 0.001	Cost analysis	CG: USD 14.51~14.51 ppp mean antibiotic prescription cost; 15.85% total prescription costs	EG: USD 16.85~16.85 ppp mean antibiotic prescription cost; 16.15% total prescription costs	The mean cost per contact: CG: 12.49 ± 29.35 $~12.49 ± 29.35 ppp EG: 13.03 ± 30.06 $~13.03 ± 30.06 ppp NS difference	An increase (USD 2.34~2.34 ppp) in mean antibiotic cost (*p* = 0.002) and in % of total antibiotic cost	Reject intervention
Pittenger et al., 2014 [49] USA	-A decrease of −29.4% in APR per ARI episode (absolute decrease −16.5 %points, 95% CI: −20.5; −12.5; *p* < 0.001) -A decrease of −9.4% in number of ARI episodes (*p* = 0.25) -A decrease of −17.0% in ARI primary care visits (*p* = 0.035)	Cost analysis	NR	NR	Cost of academic detailing per year was USD 35,192 (33,315; 37,069)~35,192 (33,315; 37,069) ppp	Cost savings related to the intervention -from the payer perspective: -avoided antibiotic prescription per year: USD 21,539 (16,317; 26,763)~21,539 (16,317; 26,763) ppp -total annual cost: USD 178,000~178,000 ppp -from the healthcare perspective: -visits avoided per year: USD 156,806 (152,358; 160,384)~156,806 (152,358; 160,384) ppp -Antibiotic costs avoided per year: USD 21,539 (16,317; 26,763)~21,539 (16,317; 26,763) ppp	Favor intervention
Schwartz et al., 2021 [2] Canada	Total antibiotic prescriptions (Relative risk): -IG1 (mailed letter on antibiotic initiation) versus CG (no letter): 0.96 (0.92; 1.01), *p* = 0.06 -IG2 (mailed letter on antibiotic duration) versus CG: 0.95 (0.91; 1.00), *p* = 0.01 -IG1 versus IG2: 0.99 (0.96; 1.02), *p* = 0.42 -IG1 and EG2 versus CG: 0.96 (0.92; 1.00), *p* = 0.02 Prolonged-duration prescriptions (>7 days) (Relative risk): -IG1 versus CG: 0.98 (0.93; 1.03), *p* = 0.42 -IG2 versus CG: 0.92 (0.87; 0.97), *p* < 0.001 -IG1 versus IG2: 0.94 (0.90; 0.98), *p* = 0.001 -IG1 and IG2 versus CG: 0.95 (0.91; 1.00), *p* = 0.02	Cost analysis	NR	Antibiotic costs (Relative risk): -IG1 versus CG: 0.97 (0.92; 1.02), *p* = 0.19 -IG2 versus CG: 0.94 (0.89; 0.99), *p* = 0.01 -IG1 versus IG2: 0.97 (0.93; 1.00), *p* = 0.03 -IG1 and IG2 versus CG: 0.96 (0.91; 1.00), *p* = 0.03	NR	The initiation letter (IG1) had no statistically significant effect. Compared with CG, the duration letter (IG2) resulted in 42 fewer antibiotic prescriptions, 24 fewer prolonged-duration prescriptions, and CAD 771~599.07 ppp in drug cost savings on average per PCP over 12 months.	Favor intervention
Walker et al., 2004 [50] USA	In 1998: 1.17 of antibiotics; 13.6% of total drugs volume In 1999: 1.08 of antibiotics; 12.1% of total drugs volume A reduction of −8.1%.	Cost analysis	In 1998: USD 19.38~19.38 ppp mean antibiotic cost per claim; 7.9% of total cost; USD 16.46~16.46 ppp mean antibiotic cost per prescription	In 1999: USD 15.09~15.09 ppp mean antibiotic cost per claim; 6.1% of total cost; USD 14.04~14.04 ppp mean antibiotic cost per prescription	NR	The average antibiotic cost per claim decreased 14.7%; The average antibiotic cost per claim decreased 22.1%; The decrease in the cost per claim for antibiotics resulted from an increase in the use of generic first-line antibiotics	Favor intervention
Wei et al., 2017 [61] China	IG (educational intervention) versus CG: -a reduction of −30% (−43 to −17) in the APR; -an increase of 2% (−1 to 5) in the multiple APR; -an increase of 5% (−10 to 20) in broad-spectrum APR; -a reduction of −8% (−20 to 5) in the intravenous APR.	Cost effectiveness analysis	CG: Antibiotic cost > Individual-Baseline: USD 0.5 (0.4)~0.5 (0.4) ppp Endline: USD 0.5 (0.4)~0.5 (0.4) ppp Cluster–Baseline: USD 0.7 (0.07)~0.7 (0.07) ppp Endline: USD 0.7 (0.06)~0.7 (0.06) ppp	IG: Antibiotic cost > Individual-Baseline: USD 0.6 (0.4)~0.6 (0.4) ppp Endline: USD 0.3 (0.4)~0.3 (0.4) ppp Cluster–Baseline: 0.7 (0.04)$~0.7 (0.04) ppp Endline: USD 0.7 (0.05)~0.7 (0.05)	NR	No significant effect of the intervention on the full prescription cost [adjusted mean difference: 0.01 (−0.03 to 0.05)] The mean antibiotic cost was significantly lower in the IG than in CG, although the crude results showed no significant difference	Reject intervention
Wei et al., 2019 [62] China	After the intervention (Antimicrobial stewardship programme > -Reduction in the APR of −49%points (95% CI: −63; −35, *p* < 0.0001); -A modest reduction in the broad-spectrum APR (−12%points (95% CI: −21; −4); After the follow-up> -Reduction in the antibiotic prescription rate of −36% points (95% CI: −55; −17, *p* < 0.0001); -A moderate reduction in the broad-spectrum APR (−20% points (95% CI: −34; −6).	Cost effectiveness analysis	Antibiotic cost in CG Baseline: USD 0.5 (±0.4)~0.5 (±0.4) ppp Post intervention: USD 0.5 (±0.4)~0.5 (±0.4) ppp Post follow-up: USD 0.5 (±0.4)~0.5 (±0.4) ppp	Antibiotic cost in IG Baseline: USD 0.6 (±0.4)~0.6 (±0.4) ppp Post intervention: USD 0.3 (±0.4)~0.3 (±0.4) ppp Post follow-up: USD 0.4 (±0.4)~0.4 (±0.4) ppp	NR	-After the intervention, a reduction in the cost of antibiotics per prescription of −0.35 (95% CI: −0.45; −0.25)$~−0.35 (95% CI: −0.45; −0.25) ppp -After the follow-up, a reduction in the cost of antibiotics of −0.26 (95% CI: −0.38; −0.13)$~−0.26 (95% CI: −0.38; −0.13) ppp	Favor intervention
Wensing et al., 2004 [65] Germany	-APR EG> baseline: 83.1%; post-intervention: 76.7% CG> baseline: 86.1%; post-intervention: 75.8% -Recommended Antibiotics EG> baseline: 46.3%; post-intervention: 47.2% CG> baseline: 43.6%; post-intervention: 44.6%	Cost analysis	Antibiotic costs per prescription: CG> baseline: EUR 21.6~24.69 ppp Post-intervention: EUR 20.9~23.89 ppp	Antibiotic costs per prescription: EG> baseline: EUR 22.5~25.71 ppp Post-intervention: EUR 21.2~24.23 ppp	NR	The intervention effect on antibiotic cost was a decrease of EUR −0.92~1.05 ppp (*p* < 0.20)	Favor intervention
Zang et al., 2018 [63] China	CG> APR: 70 (SD: 14); 4.79 (SD: 1.64) of total healthcare cost; EG> APR: 40 (SD: 19); 5.16 (SD: 1.94) of total healthcare cost; Difference of −29 (95% CI: −42; −16, *p* < 0.001) in APR and 1.02 (95% CI: −0.36; 2.4; *p* > 0.05)	Cost effectiveness analysis	Cost per %point decrease in APR in CG: USD 4.83~4.83 ppp	Cost per %point decrease in APR in EG > USD 5.33~5.33 ppp Incremental cost per percentage point reduction in APR: USD 1.02 (−0.36; 2.4)~1.02 (−0.36; 2.4) ppp	USD 390.65 (SD: 145.68)~390.65 (SD 145.68) ppp per facility, including doctors training and information resources for patients	The APR in the IG reduced by 29.23% points at an additional cost of USD 1.02 (−0.36; 2.4)~1.02 (−0.36; 2.4) ppp per patient compared to the CG, producing an ICER of USD 0.03~0.03 ppp per %point reduction in APR	Favor intervention

NS, non-significant; CI, confidence interval; +, plus; SD, standard deviation; Quality-adjusted life years (QALYs); CRP, C-reactive protein; %, percentage; APR, antibiotic prescription rate; IG, intervention group; CG, control group; DDD, defined daily doses; ppp, purchasing power parities; NA, not available; GP, general practitioner.

## Data Availability

Not applicable.

## Supplementary Materials

The following supporting information can be downloaded at: https://www.mdpi.com/article/10.3390/antibiotics11091186/s1, Table S1: PRISMA checklist; Table S2: Quality assessment of the studies included using the Joanna Briggs Institute critical appraisal checklist for economic evaluations; Table S3: Characteristics of studies selected for full data extraction (*n* = 33). Text S1: Full search expression for each database.
