# Supplementary material for: Educational Interventions to Reduce Prescription and Dispensing of Antibiotics in Primary Care: A Systematic Review of Economic Impact
Source: Antibiotics (Basel). 2022 Sep 2;11(9):1186. doi: 10.3390/antibiotics11091186 (PMC9495011; doi:10.3390/antibiotics11091186)
Supplement: Supplementary file 1 [file antibiotics-11-01186-s001.zip › antibiotics-1832469-supplementary.pdf]

Supplementary material

Table S1: PRISMA checklist

	Reporting Item	Page Number
**Title**		
Title	Identify the report as a systematic review	1
**Abstract**		
Abstract	Report an abstract addressing each item in the PRISMA 2020 for Abstracts checklist	1
**Introduction**		
Background/rationale	Describe the rationale for the review in the context of existing knowledge	1-2
Objectives	Provide an explicit statement of the objective(s) or question(s) the review addresses	2
**Methods**		
Eligibility criteria	Specify the inclusion and exclusion criteria for the review and how studies were grouped for the syntheses	2-3
Information sources	Specify all databases, registers, websites, organisations, reference lists, and other sources searched or consulted to identify studies. Specify the date when each source was last searched or consulted	2-3
Search strategy	Present the full search strategies for all databases, registers, and websites, including any filters and limits used	2-3
Selection process	Specify the methods used to decide whether a study met the inclusion criteria of the review, including how many reviewers screened each record and each report retrieved, whether they worked independently, and, if applicable, details of automation tools used in the process	2-3
Data collection process	Specify the methods used to collect data from reports, including how many reviewers collected data from each report, whether they worked independently, any processes for obtaining or confirming data from study investigators, and, if applicable, details of automation tools used in the process	3
Data items	List and define all outcomes for which data were sought. Specify whether all results that were compatible with each outcome domain in each study were sought (for example, for all measures, time points, analyses), and, if not, the methods used to decide which results to collect	3
Study risk of bias assessment	Specify the methods used to assess risk of bias in the included studies, including details of the tool(s) used, how many reviewers assessed each study and whether they worked independently, and, if applicable, details of automation tools used in the process	3
Effect measures	Specify for each outcome the effect measure(s) (such as risk ratio, mean difference) used in the synthesis or presentation of results	3
Synthesis methods	Describe the processes used to decide which studies were eligible for each synthesis (such as tabulating the study intervention characteristics and comparing against the planned groups for each synthesis (item #5))	3
Synthesis methods	Describe any methods required to prepare the data for presentation or synthesis, such as handling of missing summary statistics or data conversions	3
Synthesis methods	Describe any methods used to tabulate or visually display results of individual studies and syntheses	3
Synthesis methods	Describe any methods used to synthesise results and provide a rationale for the choice(s). If meta-analysis was performed, describe the model(s), method(s) to identify the presence and extent of statistical heterogeneity, and software package(s) used	3
Synthesis methods	Describe any methods used to explore possible causes of heterogeneity among study results (such as subgroup analysis, meta-regression)	3

Synthesis methods	Describe any sensitivity analyses conducted to assess robustness of the synthesised results	3
Reporting bias assessment	Describe any methods used to assess risk of bias due to missing results in a synthesis (arising from reporting biases)	3
Certainty assessment	Describe any methods used to assess certainty (or confidence) in the body of evidence for an outcome	3
Data items	List and define all other variables for which data were sought (such as participant and intervention characteristics, funding sources). Describe any assumptions made about any missing or unclear information	3
**Results**		
Study selection	Describe the results of the search and selection process, from the number of records identified in the search to the number of studies included in the review, ideally using a flow diagram (http://www.prisma-statement.org/PRISMAStatement/FlowDiagram)	4
Study selection	Cite studies that might appear to meet the inclusion criteria, but which were excluded, and explain why they were excluded	4
Study characteristics	Cite each included study and present its characteristics	5
Risk of bias in studies	Present assessments of risk of bias for each included study	4
Results of individual studies	For all outcomes, present for each study (a) summary statistics for each group (where appropriate) and (b) an effect estimate and its precision (such as confidence/credible interval), ideally using structured tables or plots	5-8
Results of syntheses	For each synthesis, briefly summarise the characteristics and risk of bias among contributing studies	5-8
Results of syntheses	Present results of all statistical syntheses conducted. If meta-analysis was done, present for each the summary estimate and its precision (such as confidence/credible interval) and measures of statistical heterogeneity. If comparing groups, describe the direction of the effect	5-8
Results of syntheses	Present results of all investigations of possible causes of heterogeneity among study results	5-8
Results of syntheses	Present results of all sensitivity analyses conducted to assess the robustness of the synthesised results	5-8
Risk of reporting biases in syntheses	Present assessments of risk of bias due to missing results (arising from reporting biases) for each synthesis assessed	5-8
Certainty of evidence	Present assessments of certainty (or confidence) in the body of evidence for each outcome assessed	5-8
**Discussion**		
Results in context	Provide a general interpretation of the results in the context of other evidence	18-21
Limitations of included studies	Discuss any limitations of the evidence included in the review	21
Limitations of the review methods	Discuss any limitations of the review processes used	21
Implications	Discuss implications of the results for practice, policy, and future research	18-21
**Other information**		
Registration and protocol	Provide registration information for the review, including register name and registration number, or state that the review was not registered	2
Registration and protocol	Indicate where the review protocol can be accessed, or state that a protocol was not prepared	2
Registration and protocol	Describe and explain any amendments to information provided at registration or in the protocol	2
Support	Describe sources of financial or non-financial support for the review, and the role of the funders or sponsors in the review	22
Competing interests	Declare any competing interests of review authors	22
Availability of data, code, and other materials	Report which of the following are publicly available and where they can be found: template data collection forms; data extracted from included studies; data used for all analyses; analytic code; any other materials used in the review	22

Text S1. Full search expression for each database.

PUBMED – 1339 papers

(Intervention OR education* OR program OR “health promotion” OR session OR workshop) AND (antibiotic* OR antimicrobial*) AND (economic* OR cost* OR spending OR expen*) AND (“primary care” OR “primary health care” OR “Community Health Services” OR “general practitioner” OR practitioner OR “Community Pharmacy Services” OR “Pharmaceutical services”)

SCOPUS – 1044 papers

TITLE-ABS-KEY ((intervention OR education* OR program OR "health promotion" OR session OR workshop) AND (antibiotic* OR antimicrobial*) AND (economic* OR cost* OR spending OR expen*) AND ("primary care" OR "primary health care" OR "Community Health Services" OR "general practitioner" OR practitioner OR "Community Pharmacy Services" OR "Pharmaceutical services"))

ISI web of Science – 733 papers

ALL FIELDS (Intervention OR education* OR program OR “health promotion” OR session OR workshop) AND (antibiotic* OR antimicrobial*) AND (economic* OR cost* OR spending OR expen*) AND (“primary care” OR “primary health care” OR “Community Health Services” OR “general practitioner” OR practitioner OR “Community Pharmacy Services” OR “Pharmaceutical services”)

EMBASE - 1083

(Intervention OR education$ OR program OR health promotion OR session OR workshop).af. (antibiotic$ OR antimicrobial$).af. (economic$ OR cost$ OR spending OR expen$).af. (primary care OR primary health care OR Community Health Services OR general practitioner OR practitioner OR Community Pharmacy Services OR Pharmaceutical services).af. 1 and 2 and 3 and 4

Table S2. Quality assessment of the studies included using the Joanna Briggs Institute critical appraisal checklist for economic evaluations.

**Included studies**	**Quality criterions**										
**Reference, year**	1 - Is there a well-defined question/ objective?	2 - Is there comprehensive description of alternatives?	3 - Are all important and relevant costs and outcomes for each alternative identified?	4 - Has clinical effectiveness been established?	5 - Are costs and outcomes measured accurately?	6 - Are costs and outcomes valued credibly?	7 - Are costs and outcomes adjusted for differential timing?	8 - Is there an incremental analysis of costs and consequences?	9 - Were sensitivity analyses conducted to investigate uncertainty in estimates of cost or consequences?	10 - Do study results include all issues of concern to users?	11 - Are the results generalizable to the setting of interest in the review?
Aksoy et al. (2021)	✓	✓	✓	?	✓	✓	✓	✓	✗	✓	✓
Armstrong et al. (2001)	✓	✓	✓	✓	✓	✓	✗	✓	✗	✓	✓
Balcioğlu et al. (2017)	✓	✓	✓	✓	✓	✓	N/A	✓	✗	✓	✓
Butler et al. (2012)	✓	✓	✓	✓	✓	✓	✓	✓	✗	✓	✓
Calls et al. (2011)	✓	✓	✓	✓	✓	✓	✓	✓	✓	✓	✓
Chazan et al. (2007)	✓	✓	✗	✗	✓	?	✓	✓	✗	✓	✓
Coenen et al. (2004)	✓	✓	✓	✗	✓	✓	✓	✓	✓	✓	✓
Conklin et al. (2009)	✓	✓	✓	✓	✓	✓	?	✓	✗	✓	✓
Corvoisier et al. (2013)	✓	✓	✓	✓	✓	✓	✓	✓	✓	✓	✓
Dekker et al. (2019)	✓	✓	✓	✓	✓	✓	✓	✓	✓	✓	✓
Farris et al. (1996)	✓	✓	✓	✗	✓	✓	✓	✓	✗	?	✓
Figueiras et al. (2020)	✓	✓	✓	✓	✓	✓	✓	✓	✗	✓	✓
Furst et al. (2015)	✓	✗	✓	✗	✗	✓	✓	✓	✗	✓	✓
Gillespie et al. (2016)	✓	✓	✓	✓	✓	✓	✓	✓	✗	✓	✓

Gong et al. (2019)	✓	✓	✓	✓	✓	✓	?	✓	✓	✓	✓
Hux et al. (1999)	✓	✗	✓	?	✓	✓	?	✓	✗	?	✓
Lanbeck et al. (2019)	✓	✓	✓	✗	✓	✓	✓	✓	✓	✓	✓
Madridejos-Mora et al. (2004)	✓	✓	✓	✓	✓	✓	✓	✓	✗	✓	✓
March-Lopez et al. (2021)	✓	✓	✓	✗	✓	✓	✓	✓	✗	✓	✓
Mcnulty et al. (2000)	✓	✓	✗	✗	✓	✓	✓	✓	✗	✓	✓
Me-Emary et al. (2009)	✓	✓	✓	✓	✓	✓	✓	✓	✗	✓	✓
Michaelidis et al. (2015)	✓	✓	✓	✓	✓	✓	✓	✓	✓	✓	✓
Naughton et al. (2009)	✓	✓	✓	✓	✓	✓	✓	✓	✗	✓	✓
O'Connor et al. (1999)	✓	✗	✗	✗	✓	✓	✓	✓	✗	✗	✓
Oppong et al. (2018)	✓	✓	✓	✓	✓	✓	✓	✓	✓	✓	✓
Ornstein et al. (1999)	✓	✓	✓	✗	✓	✓	?	✓	✗	✗	✓
Pittenger et al. (2014)	✓	✓	✓	✓	✓	✓	✓	✓	✓	✓	✓
Schwartz et al. (2021)	✓	✓	✓	✗	✓	✓	✓	✓	✓	✓	✓
Walker et al. (2004)	✓	✓	✓	✗	✓	✓	✓	✓	✗	✓	✓
Wei et al. (2017)	✓	✓	✓	✓	✓	✓	✓	✓	✓	✓	✓
Wei et al. (2019)	✓	✓	✓	✓	✓	✓	✓	✓	✓	✓	✓
Wensing et al. (2004)	✓	✓	✓	✗	✓	✓	✓	✓	✗	✓	✓
Zhang et al. (2018)	✓	✓	✓	✓	✓	✓	✓	✓	✓	✓	✓

Legend: ✓, Yes; ✗, No; **?,** Unclear; **N/A,** Not applicable.

Table S3: Characteristics of studies selected for full data extraction (n=33).

Author year, country	Study design	Study population	Disease	Type of intervention	Brief description of intervention	Outcome variables	Time period (months)	Perspective assessed
Aksoy et al. 2021 Turkey	Longitudinal study	Family physicians	NR	Provincial Rational Use of Medicines Coordinatorship	-Legislation reminders to prevent without prescription antibiotics<br>- Family physicians prescriptions monitoring<br>-Country-wide educations toward healthcare professionals<br>-Raising awareness letters to all family physicians<br>-Obligatory sessions on rational use of antibiotics in scientific meetings<br>-Activities to increase public awareness of rational drug use (media campaigns, advertisements, announcements, banners)<br>-Scientific activities (national and international meetings and workshops on rational drug use, regional education activities, local meetings for primary care, publication of a monthly online e-bulletin for family physicians, yearly surveillance reports on rational drug use, and scientific manuscripts)	-Percentage of antibiotic prescriptions<br>- Percentage of antibiotic items<br>- Percentage of antibiotic boxes<br>-Percentage of antibiotic cost	96	NR Healthcare service perspective
Armstrong 2001 USA	Pre post study	Physicians	Kidney & bladder infections	Guideline implementation	Promotion of the treatment guideline trough mailings and face-to-face educational interventions among physicians	-Use of antibiotics<br>-Health event costs	24	Managed care organization perspective
Balcioğlu et al. 2017 Turkey	RCT	34 Family physicians	URTI	Algorithm implementation	Algorithm based on Centor and McIsaac criteria including clinical symptoms of viral infections. Physicians of algorithm group were advised to start antibiotic treatment in scores above 60.	Differences in therapeutic approaches and treatment costs were compared in algorithm using and not using physician's groups	NR	NR Healthcare service perspective
Butler et al. 2012 UK	RCT	68 GPs	RTI	STAR educational programme	A flexible intervention to clinicians combining various learning methods and topics on antibiotic dispensing and resistance (own practice reflection, new research evidence and guidelines, video-rich material on novel communication skills, practice in usual clinical contexts, sharing experiences and views on a web form, and participating in a facilitator led, practice-based seminar).	-Total number of dispensed oral antibiotic items per 1000 patients<br>-Hospital admission rates, re-consultation rates, and costs	12	Healthcare service perspective and all costs are in 2009 prices
Cals et al. 2011 Netherlands	cRCT	40 GPs	LRTI	-CRP group<br>-Communication skills training group<br>-CRP+ communication skills training group<br>-Usual care	-The CRP intervention groups received a 30-min., practice-based introduction on the usefulness of CRP point of care testing in general practice.<br>-The communication skills training intervention was practice-based and included peer review of transcripts of consultations with simulated patients and were developed based on a patient centred strategy for shared decision making on common infections investigation and treatment.	-APR<br>-Antibiotic prescription during the 28-day follow-up period, re-consultation, patient satisfaction, enablement and clinical recovery	1	Healthcare perspective

Chazan et al. 2007 Israel	Prospective randomized study	Physicians	NR	Educational meetings	-Continuous intervention: group education meeting focusing on practical diagnostic tools to decide whether treat or not with antibiotics once a month for 2 years<br>- Seasonal intervention: a massive educational campaign - a 2-hours interactive meeting, each participant received informative reminders for themselves and educational leaflets for their patients, once a month for 2 years	-Total antibiotic use<br>-Narrow vs. broad spectrum antibiotic use<br>- Cost saving antibiotic related	24	NR<br>Healthcare service perspective
Coenen et al. 2004 Netherlands	cRCT	85 GPs	Acute cough	Tailored professional intervention	The intervention consisted in a clinical practice guideline for the management of acute cough in general practice, an educational outreach visit to GPs based on the principles of academic detailing, and a postal reminder of the key messages.	-APR<br>-Type of antibiotic prescribed<br>-effect of change in antibiotic prescription in symptom resolution<br>-Medication cost per patient	15	National Sickness and Invalidity Insurance Institute perspective
Conklin et al. 2009 Pennsylvania	Pre post study	Physicians	NR	Generic drug sampling kiosk plus academic detailing service	The kiosk is a free-standing unit approximately the size of a bank automated teller machine that dispenses generic samples in a 30-day supply or antibiotics for a complete treatment cycle.<br>Academic detailing provides the prescribers with education on benefit structures, feedback on prescription patterns, and discussions on the impact of prescription habits on member out-of-pocket cost, compliance, and adherence.	-APR<br><br>-Drug costs	24	NR<br>Managed care organization perspective
Dekker et al. 2018 Netherlands	cRCT	40 GPs	RTI	Online training	Consisted of (i) general background on the relevance of prudent antibiotic use and information about antibiotic-related problems; (ii) child-specific information on respiratory tract infections disease severity, risk factors, signs and symptoms, and the first and second choice antibiotic treatment advised; and (iii) training in enhanced communication skills.	-Antibiotic prescription rates<br><br>-Costs related to respiratory tract infection	0.5	Societal perspective and Health care perspective
Farris et al. 1996 USA	Pre post study	2 Family practice physicians	Otitis media, sinusitis, community acquired pneumonia & bronchitis	Educational intervention program	-Advanced posted notices in clinics<br>-Face to face group counter sessions including feedback of prescription trends<br>-Summary poster displays<br>-Written presentation summaries<br>-Periodic poster reminders on quality issues and costs<br>-Report of program results	-APR<br>-Net savings for each educational intervention	8	Health Maintenance Organization perspective
Figueiras et al. 2020 Spain	cRCT	3673 Physicians	NR	Multifaceted intervention	Consisting of an outreach visit, an online course, an internet-based clinical decision support system, and an information leaflet for patients.	Prescription quality<br><br>Costs of prescription	48	NHS perspective
Furst et al. 2015 Slovenia	Retrospective pre post study	Physicians	NR	Multifaceted educational programs	2-day symposium on antibiotics once a year; Prescription restrictions for amoxicillin/clavulanic acid, fluoroquinolones, macrolides and cephalosporins; Workshops in primary health centres; Informative budget targets for prescribed drugs; Guidelines on the treatment of infectious diseases; Audits; The drug bulletin 'Recept'; Workshop on	Antibiotic use<br><br>Antibiotic resistance<br><br>Costs of antibiotics	84	NR<br>Healthcare service perspective

					rational prescription of Antibiotics; Workshop in the region(s) with the highest use of antibiotics; Quality indicators including antibiotics; Antibiotic Awareness Day.			
Gillespie et al. 2016 Ireland	RCT	GPs	UTI	SIMPle intervention - multifaceted complex intervention	-Arm A: interactive workshop; national antimicrobial prescription guidelines for urinary tract infections.<br>-Arm B: interactive workshop; national antimicrobial prescription guidelines for urinary tract infections; reminder prompts outlining the prescription guidelines; monthly audits of their monthly prescription by email.	-% of antimicrobial prescriptions<br>-Antimicrobial prescriptions cost<br>-Total cost of intervention	6	Primary healthcare provider perspective
Gong et al. 2019 USA	Longitudinal study	Physicians	ARI: Acute otitis media, sinusitis & pharyngitis	Educational interventions:<br>-Suggested Alternatives (SA)<br>- Accountable Justifications (JA)<br>- Peer Comparison (PC)	SA uses computerized clinical decision support to suggest non-antibiotic treatment choices in lieu of antibiotics;<br>JA prompts entry of free-text justification that become part of the patient's electronic health record when antibiotics are prescribed;<br>PC sends a periodic email to prescribers about his/her rate of inappropriate antibiotic prescription relative to colleagues.	-Costs related to intervention implementation, provider office visit, over the counter, and symptomatic treatment for ARI;<br>-Average antibiotic cost;<br>-QALYs;<br>-Total costs.	NR	US societal perspective
Hux et al. 1999 Canada	RCT	250 Primary care physicians	NR	Confidential prescriber feedback and education	Mailed packages of prescription feedback and guidelines-based educational materials, sent every 2 months for 6 months.	-Median antibiotic cost<br>-Changes in drugs cost across intervention	6	NR Healthcare service perspective
Lanbeck et al. 2016 Sweden	Non-RCT	Physicians	NR	Individual audit-based antibiotic stewardship intervention	All audits were performed as real-time discussions, including feedback, between the auditing specialist and the ward physician	-Cost of intervention<br>-Cost of antibiotic treatment	5	NR Healthcare service perspective
Le Corvoisier et al. 2013 France	RCT	171 GPs	ARI	Educational seminar	Evidence based medicine (EBM) group: 2-day didactic educational seminar focusing on evidence-based guidelines for diagnosis and treatment of disease.<br>EBM+ problem solving strategies group: an additional 7h didactic seminar that focused on motivational enhancement and problem-solving strategies.	Changes in:<br>-antibiotic prescriptions in 2005;<br>-antibiotic prescriptions in the following years (2006, 2007);<br>-prescriptions with a symptomatic drug for respiratory infections; Cost of prescriptions;<br>Antibiotic prescription.	36	National Health Insurance System perspective
Madridejos-Mora et al. 2004 Spain	Non-RCT	282 Physicians	NR	Individualized feedback	Individualized feedback information personally delivered to each physician considering their prescription results as well as any appropriate recommendations to improve prescription quality	-Antibiotics over prescription;<br>-Antibiotics cost (euros/inhabitant)	6	NR Healthcare service perspective

March-Lopez et al. 2020 Spain	Non-RCT	208 Physicians	Pharyngotonsillitis, acute otitis media, acute sinusitis, acute bronchitis, and UTI	Antibiotic stewardship Program (ASP)	-Commitment to the ASP: 30min. face-to-face sessions directed to all relevant stakeholders (management, center directors, general physicians, pediatricians, and emergency physicians) by 25 sessions and 150 poster reminders distributed among the physicians' offices and the emergency areas.<br>-Actions for improving antibiotic prescription: a rapid test for detecting Streptococcus pyogenes and an accompanying protocol based on the Centor clinical scoring scale.<br>-Tracking and feedback: every 3-months, physicians received an updated anonymized report containing qualitative and quantitative indicators on antibiotic consumption in comparison to the previous year. Data were presented as an interactive clinical workshop.<br>-Education and experience: a set of local guidelines on antibiotic usage and preferred regimens for adult and pediatric patients.	-Antibiotic consumption;<br>-Consumption of narrow-spectrum antibiotics;<br>-Direct costs associated to antibiotic consumption	6	NR Healthcare service perspective
McNulty et al. 2000 UK	Non-RCT	GPs	NR	Workshops	12 antibiotic workshops over a 7-week period to introduce the new antibiotic guidelines, to reduce antibiotic use, to encourage the use of narrow-spectrum agents, and to improve communication with GPs.	-Antibiotics use<br>-Antibiotics cost<br>-Broad-spectrum antibiotics<br>-Narrow-spectrum antibiotics	8	NR Healthcare service perspective
Me'emary et al. 2009 Syria	Longitudinal study	76 GPs	RTI	Practical approach lung health guidelines	Patient-centred approach to the diagnosis and treatment of common respiratory illnesses encountered in primary healthcare. It promotes a symptom-based and integrated approach to management and seeks to standardize service delivery through the development and implementation of clinical guidelines.	-APR (%)<br>-Patients prescribed antibiotics per patients prescribed drugs<br>-Total cost of antibiotics (%)<br>-Total cost drug prescriptions	NR	NR Healthcare service perspective
Michaelidis et al. 2015 USA	RCT	Physicians	Acute uncomplicated bronchitis	-Printed decision support (PDS)<br>-Computerized decision support (CDS)	PDS often uses an algorithmic approach to the antibiotic prescription while the CDS strategy is integrated into the workflow of an electronic health record.	-Number of antibiotic prescriptions<br>-Costs of antibiotics<br>-Days of work loss<br>-Hospitalization within 30-days<br>-Incremental cost-effectiveness ratio	60	Societal perspective
Naughton et al. 2008 Ireland	RCT	98 GPs	NR	-Postal bulletin (PB)<br>-Academic detailing (AD)	-PB received individualized prescription feedback for 12 months prior to the intervention on rate of overall antibiotic prescription and proportion of first-line antibiotic prescription compared with second-line;<br>-AD received PB + 15-30min. outreach visit from the research coordinator consisting of a PowerPoint presentation repeating the individualized prescription feedback followed by an interactive discussion on ways of reducing antibiotic prescription.	-Volume of antibiotic prescription<br>-Proportion of first and second-line antibiotics	24	Department of Health and Children Cost perspective
O'Connor et al. 1999 USA	Pre post study	Physicians	URTI	Guideline implementation	The guideline recommends that eligible patients with respiratory symptoms be initially assessed by telephone, using a carefully constructed clinical algorithm. The guideline strongly recommends against the use of antibiotics in URTI	-Proportion of patients with antibiotics<br>-Cost of initial care	1	NR Healthcare service perspective

Oppong et al. 2018<br>Belgium, Netherlands, Poland, Spain, UK	cRCT	Physicians	NR	-CRP group<br>-Communication group<br>-CRP+ communication skills group<br>-Usual care	-The CRP intervention consisted in internet training on how to target testing and how to negotiate with the patient on management decisions.<br>-The communication skills intervention consisted of patients' data on concerns and expectations, exchange of information on symptoms, natural disease course, and treatments, agreement of a management plan, summing up, and providing guidance about when to reconsult. Physicians were also provided with an interactive booklet to use during consultations that included information on symptoms, use of antibiotics and antibiotic resistance, self-help measures, and when to re-consult.	-APR<br>-Cost<br>-QALYs<br>-Incremental cost-effectiveness ratio	1	Healthcare service perspective<br><br>All cost in 2016 prices
Ornstein et al. 1999<br>USA	Non-RCT	Family physicians	NR	-Prescriptions without cost information<br>-Drug cost information at the time of prescription group	A computer-based patient record system on prescription provided brand name and generic cost data for prescriptions. Less expensive alternatives in the same pharmacological class could be also displayed. Cost information was only displayed when an exact match occurred between the medication name and dose in the prescription and CPR data dictionary.	-Prescriptions<br>-Average cost of prescription	12	NR<br>Healthcare service perspective
Pittenger et al. 2014<br>USA	Retrospective cohort study	Physicians	ARI	Academic detailing	Education on appropriate indications for treatment in ARI, tracking, and local benchmarking of provider antibiotic prescription rates. The presentations included slide presentations with summaries of the medical evidence and guidelines, as well as benchmark data on the institution and the individual providers, with trends. The presentation also included a summary of the regional Washington Health Alliance “Community Checkup,” which provided a comparison of our institution to other regional groups.	-APR;<br>-Number of ARI visits avoided<br>-Total number of ARI primary care visits<br>-Average cost of antibiotic<br>-Antibiotic cost avoided	35	Healthcare payer perspective<br><br>Healthcare delivery system perspective
Schwartz et al. 2021<br>Canada	RCT	3465 Primary care physicians	NR	Mailed letter addressing antibiotic treatment initiation;<br>Mailed letter addressing antibiotic duration;<br>No letter on antibiotic prescription	Each letter informed the physicians that they were in the highest 25th percentile compared with their peers for antibiotics prescribed based on their total number of antibiotic prescriptions. The initiation letter provided a table on appropriate antibiotic initiation for common respiratory infections. The duration letter provided recommendations on appropriate antibiotic prescription durations. The control group did not receive a letter.	-Total antibiotic volume based on number of prescriptions<br>- number of prolonged duration antibiotic prescriptions (>7 days)<br>-Total antibiotic costs	11	NR<br>Healthcare service perspective
Walker et al. 2004<br>USA	Retrospective pre post study	Pharmacists and 65 physicians	NR	Educational intervention	Combination of drug utilization analysis, academic detailing, and staff education to influence prescription.	-Average cost per claim<br>-Average cost for all prescription drug claims	24	NR<br>Healthcare service perspective
Wei et al. 2017<br>China	cRCT	571 Physicians	URTI	Training and educational intervention	Evidence-based prescription guideline, training and monthly prescription peer-review meetings for doctors, and brief education for caregivers	-APR;<br>-Multiple antibiotic prescription rate;	6	NR

					during consultations and an educational waiting room video for caregivers.	-Broad-spectrum antibiotic prescription rate;<br>-The intravenous antibiotic prescription rate;<br>-The full prescription cost;<br>-The antibiotic medication cost		Healthcare service perspective
Wei et al. 2019 China	cRCT	507 Physicians	URTI	Antimicrobial stewardship programme	Referring to our guidelines for clinical knowledge and communication skills regarding respiratory disease, conducting peer reviews of antibiotic prescription on a regular basis in existing team meetings, and providing concise education to patients on antibiotic use.	-APR;<br>-Multiple antibiotic prescription rate);<br>-Broad-spectrum antibiotic prescription rate;<br>-The intravenous antibiotic prescription rate;<br>-The full prescription cost;<br>-The antibiotic cost	6 +18 (FU)	NR Healthcare service perspective
Wensing et al. 2004 Germany	Pre post study	177 Primary care physicians	NR	Quality Circles	Repeated feedback on prescription routines and an intensive programme of educational small group sessions. In sum 10 groups of 10–12 doctors had 11 meetings of 2 hours moderated by a primary care doctor, who had received a short training. Each meeting focused on a specific group of drugs using clinical guidelines and other evidence-based information as well as a video that showed a demanding patient.	-APR<br>-Antibiotic costs per prescription<br>-Recommended antibiotics of all antibiotic prescriptions (%)	24	NR Healthcare service perspective
Zang et al. 2018 China	cRCT	Primary care physicians	URTI	Antimicrobial stewardship programme	Evidence-based clinical guidelines on URTI management, facilitated training on using/applying the guidelines during consultations and monthly peer review meetings assessing providers' antibiotic prescription rates. Patients and caregivers received information on appropriate antibiotic use, both verbally and via an educational leaflet. A video with key messages on appropriate use of antibiotics was also played daily in the waiting rooms and public areas.	-APR<br>-Total healthcare costs (consultation, prescription monitoring+review meeting, medication costs)<br>-Cost per %point decrease in APR;<br>-Incremental cost per percentage point reduction in APR;	6	Healthcare provider perspective

Legend: NR, not referred; CI, confidence interval; +, plus; SD, standard deviation; Quality-adjusted life years (QALYs); RCT, randomised controlled study; cRCT, cluster randomised controlled study; Non-RCT, non-randomized controlled study (i.e., quasi-experimental study); APR, antibiotic prescription rate; URTI, upper respiratory tract infections; ARI, acute respiratory infection; LRTI, lower respiratory tract infections; RTI, respiratory tract infections; UTI, urinary tract infection; USA, United States of America; UK, United Kingdom; NHS, National Health Service; %, percentage; GP, general practitioner;
